# Interprofessional collaboration and patient-reported outcomes: a secondary data analysis based on large scale survey data

**DOI:** 10.1186/s12913-022-08973-5

**Published:** 2023-01-03

**Authors:** Laura Kaiser, Edmund A. M. Neugebauer, Dawid Pieper

**Affiliations:** 1 Witten/Herdecke University, Witten, Germany; 2 Brandenburg Medical School Theodor Fontane, Neuruppin, Germany; 3 Institute for Research in Operative Medicine, Witten, Germany; 4 Institute for Health Services and Health Systems Research, Brandenburg Medical School Theodor Fontane, Rüdersdorf, Germany; 5 Center for Health Services Research, Brandenburg Medical School Theodor Fontane, Rüdersdorf, Germany; 6 Faculty of Health Sciences Brandenburg, Potsdam, Germany

**Keywords:** Interprofessional collaboration, Patient-reported outcomes, Patient-reported experiences, Survey, Inpatient

## Abstract

**Background:**

While interprofessional collaboration (IPC) is widely considered a key element of comprehensive patient treatment, evidence focusing on its impact on patient-reported outcomes (PROs) is inconclusive. The aim of this study was to investigate the association between employee-rated IPC and PROs in a clinical inpatient setting.

**Methods:**

We conducted a secondary data analysis of the entire patient and employee reported data collected by the Picker Institute Germany in cross-sectional surveys between 2003 and 2016. Individual patient data from departments within hospitals was matched with employee survey data from within 2 years of treatment at the department-level. Items assessing employee-rated IPC (independent variables) were included in Principal Component Analysis (PCA). All questions assessing PROs (overall satisfaction, less discomforts, complications, treatment success, willingness to recommend) served as main dependent variables in ordered logistic regression analyses. Results were adjusted for multiple hypothesis testing as well as patients’ and employees’ gender, age, and education.

**Results:**

The data set resulted in 6154 patients from 19 hospitals respective 103 unique departments. The PCA revealed three principal components (department-specific IPC, interprofessional organization, and overall IPC), explaining 67% of the total variance. The KMO measure of sampling adequacy was .830 and Bartlett’s test of sphericity highly significant (*p* < 0.001). An increase of 1 SD in department-specific IPC was associated with a statistically significant chance of a higher (i.e., better) PRO-rating about complications after discharge (OR 1.07, 95% CI 1.00–1.13, *p* = 0.029). However, no further associations were found. Exploratory analyses revealed positive coefficients of department-specific IPC on all PROs for patients which were treated in surgical or internal medicine departments, whereas results were ambiguous for pediatric patients.

**Conclusions:**

The association between department-level IPC and patient-level PROs remains – as documented in previous literature - unclear and results are of marginal effect sizes. Future studies should keep in mind the different types of IPC, their specific characteristics and possible effect mechanisms.

**Trial registration:**

Study registration: Open Science Framework (DOI 10.17605/OSF.IO/2NYAX); Date of registration: 09 November 2021.

**Supplementary Information:**

The online version contains supplementary material available at 10.1186/s12913-022-08973-5.

## Background

Health care practitioners are nowadays confronted with a steadily aging patient structure and, therefore, face interprofessional needs in the treatment of chronic and/or complex diseases. Interprofessional collaboration (IPC) is defined by the World Health Organization as a patients’ treatment in which “multiple health workers from different professional backgrounds provide comprehensive services by working with patients, their families, carers and communities to deliver the highest quality of care across settings” [1]. However, IPC “is increasingly used but diversely implemented” [2] making the assessment of health care quality difficult. Moreover, a holistic picture of care can only be drawn if, in addition to clinical quality indicators, patient-specific outcomes are included in health system performance assessments.

The overall evidence of the association of IPC with patients’ outcomes remains uncertain. Whereas positive effects have been found with regard to clinical endpoints [3], patient-reported outcomes (PROs) are underrepresented in the literature and the little data available do not show a clear direction of relationships [2–7].

In this study, we aimed to evaluate the association between employee-rated IPC and PROs in inpatient settings. Secondly, we wanted to provide an exploratory analysis of department-specific differences that were encountered.

## Methods

This study was guided by the recommendations of the Good Practice of Secondary Data Analysis (GPS, third revision 2012/2014) [8]. The pre-analysis plan for this secondary data analysis has been preregistered in the Open Science Framework Registry (DOI 10.17605/OSF.IO/2NYAX).

There are two modifications in comparison with our pre-analysis plan:

- The preplanned investigation of covariates using analyses such as lasso, elastic net, or ridge were rejected due to the ordinal scaling of item responses.
- As the addition of demographic (control) variables to the ordered logistic regression model revealed a high number of missing values, we decided to waive imputation but analyze only those patients from whom we got demographic (control) details.

### Picker survey data

The dataset was comprised of the entire patient and employee reported data that the Picker Institute Germany collected in 342 cross-sectional surveys between 2003 and 2016, which was the entire time period in which the Picker Institute conducted surveys in Germany. They were conducted on the occasion of quality improvement and monitoring processes in hospitals (i.e., the timing of the surveys was based on single occasions and the dataset does not represent systematic repeated cross-sections or panel data). For this purpose, validated employee (“Picker Employee Questionnaires”) as well as patient questionnaires (“Picker Inpatient Questionnaires”) were applied in different departments within hospitals. Good psychometric properties have been shown in several investigations [9–13]. The paper-pencil questionnaires were introduced to employees respective patients along with up to two reminders according to the Total Design Method by Dillman [14]. Initial information on voluntariness in participation and pseudonymized analysis was given (see declarations for further information on ethics approval and consent to participate). Only those patients who did not respond within two (1st reminder) or 4 weeks (2nd reminder) received an additional reminder with a copy of the questionnaire and a return envelope. As employee surveys generally result in a lower response rate, particular importance was attached to ensuring a high level of commitment and willingness to participate in these surveys. For this reason, employee surveys were conducted anonymously, which meant that all those initially contacted also received all additional reminder letters. Employees who have already answered were advised to dispose the questionnaire in the reminder letter.

### Measured variables

General information on the composition of Picker questionnaires can be found in Additional file 1. For our analyses, we used all questions assessing PROs as *main dependent variables*. Items assessing employee-rated IPC were included in Principal Component Analysis (PCA, see statistical analysis). The resulting principal components served as *independent variables* in further analyses. Demographic variables (gender, age, and education) were used as *control variables* for employees as well as patients. Items come with different response options, ranging from binary item formats to items with up to seven on a Likert scale (higher scores represent better IPC respective PRO). The specific items can be found in the Additional file 2.

### Data analysis

All calculations have been performed using Stata MP 17.0. The level of significance was defined as 0.05. As some hospitals decided to survey their patients and employees in two consecutive years for organizational reasons (instead of conducting the two types of surveys simultaneously), data from hospitals that conducted both an employee and patient survey within a two-year period were analysed. In addition, the departments surveyed had to be identical. As we were able to create unique hospital-department-codes, consisting of a unique hospital- plus a unique code for a given department, irrespective of questionnaire type (patient or employee questionnaire) or survey year, we were able to match the respective department-level IPC score to the individual-level patient data.

A PCA on items assessing IPC from the employee’s point of view was used to derive main components underlying these scales. The overview of all items included in PCA can be found, along with the prespecified PCA model, in Additional file 3. Items were identified as suitable for main components if the Kaiser-Meyer-Olkin (KMO) measure of sampling adequacy coefficient was > 0.50, and the Bartlett’s test of sphericity significant (i.e., < 0.05). Only factors with eigenvalues ≥1 were considered (Guttmann-Kaiser Criterion) and results were rotated using both promax and varimax rotation [15–17] in order to check the sensitivity of results to these alternative interpretations. Reassuringly, the results mirrored the unrotated principal solution. Thus, we rely on the unrotated solution to predict standardized component scores for the employees and subsequently aggregate these at the department-year-level (i.e., the department-year-IPC-score for each component was the mean of the employee component scores for each component).

These mean component scores were z-standardized to allow the statement that an increase of 1 SD on IPC score at department-year-level was associated with a change of *β*_1_ (OR) in PRO outcomes at the individual-patient-level. The unit of analysis was individual patient data from departments within hospitals which have surveyed employees from the identical departments in the same year, the year before or after.

Due to the ordinal scaling of the dependent variables (i.e., PROs), the regression model was implemented as an ordered logistic regression model, such that estimated coefficients (logged odds) are subsequently transformed and interpreted as odds ratios (OR). The formal model takes the general form:

$${\Pr}\left({y}_{icj}=l\right)={\Pr}\left({K}_{l-1}<{\beta}_1{IPC}_{cj}+{\beta}_k{X}_{kij}+{\beta}_g{X}_{gcj}+{\theta}_h+{\varepsilon}_{icj}\le {K}_l\right)$$

where y_icj_ denotes PRO of patient *i* treated in department *c*. As the outcome variable *y*_*j*_ consists of ordered outcome categories, the score was estimated as a linear function of the independent variables (department-level IPC and department- and individual-level covariates) and cutpoints defined by the number of categories of the dependent variable. The probability of observing outcome *l* was then defined as the probability of the function and error term being within the range of the estimated cutpoints. β_1_ was the coefficient of interest denoting the change in the probability of outcomes in response to a one-unit increase in the department-year-level IPC score (IPC_c_). *X*_*kij*_ and *X*_*gcj*_ denote vectors of individual and cluster-level control variables, respectively. θ_h_ denotes hospital fixed effects and *ε*_*icj*_ denotes the random error term. Standard errors are cluster-robust at the department-year-level. Additionally, we adjust the reported *p*-values for the false discovery rate (FDR) using Andersons’ q values for multiple hypothesis testing, and examine of the association between patient- and employee-rated IPC to assure validity of the IPC measure.

In order to answer the secondary research questions, exploratory subgroup analyses were undertaken afterwards. Results were adjusted for multiple hypothesis testing based on the false discovery rate [18].

## Results

### Population characteristics

In total, 43 Hospitals including 519 departments and 88,243 individual-level observations (25,470 employees and 62,773 patients) surveyed both employees and patients within 2 years in the period from 2003 to 2016 (*n* = 132 surveys). After PCA, matching of employee-based department-level IPC scores to patient-level PRO data, and limitation to those patients for whom demographic (control) variables were available (see below) there were 6154 patient observations (unit of analysis) from 19 hospitals respective 103 departments and 49 surveys included in the analyses. Table 1 presents employees’ and patients’ descriptive statistics. Survey characteristics, including information regarding the specific survey years, department types and number of surveyed patients and employees from each included hospital can be found in Additional file 4.

**Table 1:** Descriptive statistics

Descriptive statistics	n (%)
**Employees**	
*Female* (*n* = 16,957)	12,740 (75.1)
*Age (years) (n = 13,642)*	
18 to 24	746 (5.5)
25 to 29	1684 (12.3)
30 to 34	1785 (13.1)
35 to 44	3820 (28.0)
45 to 54	3999 (29.3)
55 to 59	1220 (8.9)
60 or older	388 (2.8)
*Educational attainment (n = 16,776)*	
No school degree	28 (0.2)
Lower track degree	1514 (9.0)
Middle track degree	7063 (42.1)
Higher education entrée degree	3166 (18.9)
Academic degree	5005 (29.8)
**Patients (*n*** **= 6154)**	
*Female*	2934 (47.7)
*Age (in years, mean)*	62.2
*Educational attainment*	
Lower track degree	1224 (19.9)
Middle track degree	3525 (57.3)
Higher education entry degree	387 (6.3)
Academic degree	1018 (16.5)

The school system in Germany has three types of general education secondary schools, tracking students by ability and leading to different exit degrees. These tracks encompass schools where students qualify to enter vocational training (e.g., lower and middle tracks), combine both vocational training with the option of attending university later (middle track), or directly focus on preparation for higher education (i.e., university) studies (Higher education entry degree)

### PCA

The PCA revealed three principal components, explaining 67% of the total variance:

- Department-specific IPC (component 1) contains four items which focus on trust, support, professional relationship, and problem solving with colleagues working within the same department,
- Interprofessional organization (component 2) includes three questions on clearly defined tasks and structuredness of team meetings or handover talks,
- Overall IPC (component 3) was comprised of two items addressing the professional relationship with colleagues from other departments.

Both promax and varimax rotation revealed the same composition of components. Thus, we rely on the original (unrotated) unrotated principal solution to predict the component scores. The KMO measure of sampling adequacy was .830 and Bartlett’s test of sphericity significant (*p* < 0.001). The final set of IPC items can be found in Additional file 2.

### PRO items

All items assessing PROs from patients which were included in the final data set were used for regression analyses (see Additional file 2 for main dependent variables).

### Association between employee-rated IPC and PRO

When neither controlling for employees’ nor patients’ demographic characteristics (gender, age, education) a statistically significant association was found between the PRO complications and department-specific IPC, meaning that an increase of 1 SD in the first IPC component leads to a statistically significant chance of a higher (i.e., better) PRO-rating regarding the question “Did complications occur after your/ your child’s discharge from hospital?” (OR 1.06, 95% CI 1.00–1.11, *p* = 0.046). This result was consistent after adding control variables to the ordered logistic regression model (OR 1.07, 95% CI 1.00–1.13, *p* = 0.029) (see Table 2).

**Table 2:** Comparison between adjusted and unadjusted results (in OR) from ordered logistic regression (*n* = 6154 patients)

	Overall satisfaction	Less discomforts	Complications	Treatment success	Willingness to recommend
**Department-specific IPC**	U: 1.03 (0.95–1.11), *p* = 0.492,	U: 1.05 (0.96–1.15), *p* = 0.288	U: 1.06 (1.00–1.11), *p* = 0.046	U: 1.05 (0.99–1.11), *p* = 0.122	U: 1.02 (0.95–1.10), *p* = 0.522
	A: 1.06 (0.98–1.12), *p* = 0.136, q = 0.64	A: 1.07 (0.96–1.19), *p* = 0.208, q = 0.64	A: 1.07 (1.00–1.13), *p* = 0.029, q = 0.64	A: 1.05 (0.98–1.12), *p* = 0.206, q = 0.64	A: 1.06 (0.99–1.12), *p* = 0.110,q = 0.64
**Interprofessional organization**	U: 1.06 (0.96–1.16), *p* = 0.235	U: 1.03 (0.94–1.12), *p* = 0.538	U: 0.94 (0.89–1.00), *p* = 0.071	U: 1.04 (0.97–1.12), *p* = 0.272	U: 1.05 (0.97–1.13), *p* = 0.256
	A: 1.03 (0.95–1.13), *p* = 0.380, q = 0.64	A: 1.03 (0.94–1.13), *p* = 0.494, q = 0.64	A: 0.95 (0.89–1.01), *p* = 0.096, q = 0.64	A: 1.03 (0.95–1.12), *p* = 0.430, q = 0.64	A: 1.04 (0.97–1.12), *p* = 0.302, q = 0.64
**Overall IPC**	U: 0.99 (0.90–1.08), *p* = 0.818	U: 0.92 (0.80–1.06), *p* = 0.263	U: 1.03 (0.96–1.12), *p* = 0.399	U: 0.99 (0.92–1.07), *p* = 0.830	U: 0.96 (0.88–1.04), *p* = 0.311
	A: 0.97 (0.88–1.07), *p* = 0.526, q = 0.64	A: 0.90 (0.79–1.02), *p* = 0.108, q = 0.64	A: 1.02 (0.96–1.09), *p* = 0.568, q = 0.64	A: 1.00 (0.92–1.08), *p* = 0.954, q = 0.803	A: 0.93 (0.85–1.03), *p* = 0.167, q = 0.64

U = unadjusted, A = adjusted; results are presented in OR with 95% CI, p = *p*-value (unadjusted for multiple inference), q = sharpened FDR-q-value based on Benjamini et al. 2006

No statistically significant results between IPC components and PROs emerge neither with nor without adding control variables in the remaining analyses (see Table 2).

To account for the problem of multiple inference (i.e., 15 tests due to 5 outcomes and three IPC variables), we also report sharpened q-values for the adjusted regression estimates based on the method developed by Benjamini et al. [19], i.e., the expected proportion of false rejections of the null hypothesis [18]. This approach allows a straightforward comparison with the original *p*-values and has more power than Bonferroni correction: With a large number of tests, Bonferroni correction (which controls for the familywise error rate, i.e.., the probability of any false rejections of the null hypothesis), may be considered too conservative. In our case, both sharpened q-values and Bonferroni corrected *p*-values (i.e., *p*-values multiplied by the number of tests) are severely inflated, indicating no significant relationship between IPC and PROs in any of our analyses when accounting for multiple inference.

### Robustness

Focusing on the association between patient- and employee-rated IPC, we estimated OR (using ordered logistic regression) regarding the question “How well does the communication and coordination between doctors and nurses work?” which was asked of patients as well as employees. Here, a statistically significant result (OR 1.10, 95% CI 1.02–1.18, *p* = 0.011) was found (*n* = 5345 patients), meaning that patients as well as employees of this sample have comparable ratings of IPC.

### Exploratory analyses

Regarding exploratory analysis, we decided to focus on heterogenous associations between IPC and PRO by department-type. Given the fact, that there are 13 department types left in the final data set, we decided to analyze the three departments with the largest sample size. As a result, we analyzed “Internal medicine” (*n* = 2100), “Surgery” (*n* = 1698), and “Pediatrics” (*n* = 442). As stated in the pre-analysis plan (see above), results generated in these exploratory analyses are intended to be viewed as hypothesis generating rather than hypotheses testing. Nevertheless, we investigated Andersons’ q values to account for the multitude of analyses.

Results are presented in the Additional file 5. Significant Andersons’ q values confirm the significance of all *p* values emerged in these analyses. Statistically significant results for “Surgery” and “Internal medicine” are found only regarding one single IPC-component (department-specific IPC). In accordance with the above-described results, an increase of 1 SD in department-specific IPC leads to higher odds for a better PRO-rating of patients which were treated within these departments, not only in relation to the PRO complications, but also to the other investigated PROs.

To the contrary, results for pediatric patients are more ambiguous. Whereas the chance of a better PRO-rating regarding complications was higher with an increase in department-specific IPC (OR 1.15, 95% CI 1.07–1.23, *p* < 0.001), it was lower with an increase in interprofessional organization (second IPC component) (OR 0.59, 95% CI 0.44–0.79, *p* < 0.001). The opposite picture arises when looking at the PROs overall satisfaction, less discomforts, and treatment success. Regarding the third IPC component (overall IPC) an increase of 1 SD was associated with a worse PRO-rating in all investigated outcomes.

## Discussion

The aim of this observational study was to investigate the association between employee-rated IPC and PROs. To answer this research question, we analyzed cross-sectional survey data of employee as well as matched patient surveys which have been conducted by the Picker Institute Germany between 2003 and 2016. After PCA, IPC was subdivided into three different components: department-specific IPC, interprofessional organization, and overall IPC. Five PRO items, focusing on overall satisfaction, the improvement of discomforts, complications, subjective overall treatment success, and willingness to recommend, were available as outcomes for analyses. After data cleaning, the final dataset contained 6154 patients from 19 hospitals respective 103 departments for whom employee-rated department-level IPC scores could be matched. Robustness analysis revealed comparable ratings of IPC confirmed that linking employee-rated IPC with patient-rated PROs was valid in terms of answering our research question. In summary, all OR revealed in the analyses are about 1 suggesting that there was neither a positive nor negative chance of better PRO rating with increasing department-specific IPC, interprofessional organization, or overall IPC (see Table 2).

As previous PRO studies often lack large-scale survey designs [2, 3, 20] the generalizability of these previous results can be questioned in many cases. In contrast, the strength of this secondary data analysis study lies in a dataset containing not only a relatively large sample size of employee and patient reports but also a multitude of hospitals and departments. Therefore, we are confident to assume a high external validity for Germany. Moreover, our study was innovative as the dataset enabled us to match data from both employee and patient surveys regarding a PRO-specific research question with a well validated PRO measure. The Picker Questionnaire was rated as “the most likely to cover important areas regarding hospital stay and [ …] the least difficult to understand as well as [ …] the best designed questionnaire” [21] in a randomized trial of four different patient satisfaction questionnaires [22].

Overall, results show marginal positive or negative OR respective effect sizes, meaning there was neither evidence of a negative nor a positive association between IPC and PRO. The patient-related outcome “complications” was the only one which was significantly associated with IPC. A positive effect, emerged when focusing on “department-specific IPC” and “complications”. However, Anderson’s q valued were insignificant in each case assuming that significant results are likely due to multiple hypothesis testing.

Regarding exploratory analyses there are some positive associations with regard to the departments surgery, and internal medicine with significant Andersons’s q values as well. Yet, contradictory results emerged when focusing on pediatrics, were IPC components show negative impacts on pro-ratings in some cases.

Other secondary data analyses of Picker survey data are not available. Nevertheless, our results are comparable to those found in experimental studies using the Picker Questionnaire for assessing PROs after specific interprofessional interventions. Luthy et al. [21] used the Picker Questionnaire to assess the effect of interprofessional bedside-rounds versus daily rounds outside the patients’ room in a quasi-experimental controlled study. Even if the intervention was found to have a positive effect on the patient-healthcare professionals’ satisfaction in terms of interprofessional collaboration, the willingness to recommend was, in fact, negatively associated with this. O’Leary et al. [5] studied a similar research question in a cluster-randomized controlled trial and used the Picker Questionnaire for the assessment of patients’ satisfaction after discharge. Here, the patients’ overall satisfaction did not differ between intervention and control group. In studies using other kinds of PRO measures the association between IPC and PROs in terms of quality of life, symptoms, anxiety, and depression remained unclear, as well [3, 4, 6]. With regard to the different effects of the above-named IPC components in our analysis, Carron et al. [2] also came to the conclusion, that the effect of IPC was likely related to the specific IPC-type.

According to Reeves et al. [23], IPC interventions can be divided into three sections: interprofessional education, interprofessional practice, and interprofessional organization. Our items (and components) do not directly map into this definition of IPC but include several items related to components also discussed in Reeves et al.: Whereas the components one (“department-specific IPC”) and three (“overall IPC”) both cover items addressing interprofessional practice, component two (“interprofessional organization”) includes items focusing on areas also covered in Reeves et al. under the same term. Unfortunately, items addressing interprofessional education are not included in any of the three components. However, in a Cochrane Review by Reeves et al. [24] positive effects of interprofessional education interventions for healthcare professionals on patient outcomes, adherence rates, patient satisfaction, and clinical process outcomes were found in several studies.

Even if the effect of IPC on specific PROs remains unclear, the effectiveness of IPC should not be defined solely on the basis of PROs but also in the light of different kinds of clinical outcomes. For example, Pascucci et al. 2021 [3] systematically reviewed the literature and found positive effects of interprofessional interventions on clinical outcomes, such as levels of systolic blood pressure, diastolic blood pressure, blood levels of Glycated Hemoglobin (HbA1c)], cholesterol level, decrease of smoking, and in the duration of hospitalization.

This study has several limitations. Firstly, we must admit that items used for analyses are not partially validated but within the validation process of the whole questionnaire. As the surveys were part of quality improvement processes within hospitals and therefore did not focus on IPC in detail, survey participants were neither advised to the definition of IPC nor was information on the nature of IPC within departments collected. However, as Picker Questionnaires were validated using focus groups as well as pilot studies to ensure content and face validity [9–13], a common understanding of IPC can be assumed. Secondly, our sample consists of hospitals which self-selected themselves into being surveyed and data represents a voluntary sample of employees and patients within a given time-frame. Details about these self-selection mechanisms are not available.

Initiatives such as PaRIS (Patient-Reported Indicator Surveys) launched by the OECD in 2017 show the future direction of PROs in the context of health system assessments [25]. In PaRIS, the aim is to develop and standardize patient-reported indicators as well as to survey PROs and patient-reported experiences of people with chronic diseases systematically in order to assess how health systems address patients’ needs [25]. It will be necessary to examine in future studies in how far results obtained from initiatives like this may allow further exploration of the effect of IPC. Moreover, future studies should discuss the conceptualization of IPC and study the effect mechanism of the different IPC types. As our study focuses on inpatients, a comprehensive overview by concentrating on the outpatient clinical setting is desirable.

## Conclusion

Our findings suggest that, overall, the association between department-level IPC and patient-level PROs remains unclear and results are of marginal effect sizes. However, PROs represent only one piece of a puzzle in the examination of IPC and promising results were found in previous studies examining the effect of IPC on clinical outcomes. As the three types of IPC may have different effect mechanisms between IPC types, the investigation of the effectiveness of IPC should keep in mind the different aspects of these types.

## Supplementary Information

**Additional file 1.** Questionnaires.

**Additional file 2.** List of variables.

**Additional file 3.** Prespecified Model for PCA.

**Additional file 4.** Survey characteristics.

**Additional file 5.** Exploratory Analyses.

## Data Availability

The analysis relies on proprietary data collected by the Picker Institute Germany up until March 2016. As such, the data are not publicly available. As the Picker Institute Germany was incorporated to the BQS Institute for Quality and Patient Safety in April 2016, the BQS is the legal entity owning the data. Thus, any requests may be directed to the BQS Institute. However, the code to generate the results (Stata Do-file/ MP 17.0) is available on request.

## Abbreviations

FDR: False discovery rate
GPS: Good Practice of Secondary Data Analysis
IPC: Interprofessional collaboration
KMO: Kaiser-Meyer-Olkin measure of sampling adequacy coefficient
OR: Odds ratio
PCA: Principal Component Analysis
PRO: Patient-reported outcomes
