# Supplementary material for: Interprofessional collaboration and patient-reported outcomes: a secondary data analysis based on large scale survey data
Source: BMC Health Serv Res. 2023 Jan 3;23:5. doi: 10.1186/s12913-022-08973-5 (PMC9809039; doi:10.1186/s12913-022-08973-5)
Supplement: Supplementary file 1 — Additional file 1. Questionnaires. [file 12913_2022_8973_MOESM1_ESM.docx]

**Additional file 1: Questionnaire composition**

The Picker Employee Questionnaire from 2015 contains 111 questions including eight questions for demographical and vocational details and two questions asking for free text commentaries. The remaining questions address, among others, experiences in interprofessional collaboration, workload, organizational structures and processes [1].

The Picker Inpatient Questionnaire from 2013 comprises 86 questions, with eight questions addressing demographic details and two open questions at the end of the survey. Except for some PRO questions on satisfaction, self-reported treatment success, etc. in the Picker Inpatient Questionnaire (see below), both questionnaires are fact-oriented, meaning that they are focusing on concrete experiences of employees or patients (PREs, i.e., “Did doctors talked about you in your presence as if you were not there?”).

Because of questionnaire development processes which occurred between 2003 and 2016 the number of items per questionnaire version varies to some extent. However, the majority of items is asked in all versions. Additionally, we included only those items that were administered in all of the departments in the final sample.

Items come with different response options, ranging from binary item formats to items with up to five on a Likert scale in the Picker Employee, and up to six in the Inpatient Questionnaire. Where it made sense in terms of content, the option of answering with "Does not apply" was also given [2].

--

Reference:

1. Stahl K, Schirmer C, Kaiser L. Adaption and Validation of the Picker Employee Questionnaire With Hospital Midwives. J Obstet Gynecol Neonatal Nurs. 2017;46:e105–17.

2. Riechmann M, Stahl K. [Employee satisfaction in hospitals - validation of the Picker employee questionnaire: the German version of the “survey of employee perceptions of health care delivery” (Picker Institute Boston)]. Gesundheitswesen. 2013;75:e34-48.
