# Supplementary material for: Interprofessional collaboration and patient-reported outcomes: a secondary data analysis based on large scale survey data
Source: BMC Health Serv Res. 2023 Jan 3;23:5. doi: 10.1186/s12913-022-08973-5 (PMC9809039; doi:10.1186/s12913-022-08973-5)
Supplement: Supplementary file 2 — Additional file 2. List of variables. [file 12913_2022_8973_MOESM2_ESM.docx]

**Additional file 2: Variables**

Table 1: List of control variables

| Label | Item | Scale |
| --- | --- | --- |
| Picker Employee Questionnaire | | |
| Gender | You are... | A woman (1) – a man (2) |
| Age | What is your age group? | 18 to 24 years (1) – 25 to 29 years (2) – 30 to 34 years (3) – 35 to 44 years (4)– 45 to 54 years (5) – 55 to 59 years (6)– 60 years or older (7) |
| Education | Which general or vocational qualification do you have or are you aiming for? | No school degree (1) Lower track degree (2) Middle track degree (3) Higher education entree degree (4) Academic degree (5) |
| Picker Inpatient Questionnaire | | |
| Gender | You are... | female (1) – male (2) |
| Age | How old are you? | continuous |
| Education | Which general or vocational qualification do you have or are you aiming for? | Lower track degree (1) Middle track degree (2) Higher education entree degree (3) Academic degree (4) |

Note: The school system in Germany has three types of general education secondary schools, tracking students by ability and leading to different exit degrees. These tracks encompass schools where students qualify to enter vocational training (e.g., lower and middle tracks), combine both vocational training with the option of attending university later (middle track), or directly focus on preparation for higher education (i.e., university) studies (Higher education entry degree).

Table 2: Items assessing employee-rated IPC (independent variable)

| Item | Scale |
| --- | --- |
| Component 1: Department-specific IPC | |
| Do you trust the colleagues, you work with directly? | 1 No – 2 Partly – 3 Mostly – 4 Yes, completely |
| Do you get necessary support from your colleagues? | 1 Rarely – 2 Partly – 3 Mostly – 4 Always |
| Are you treated by colleagues in your own ward/ your department as you wish? | 1 Rarely – 2 Partly – 3 Mostly – 4 Always |
| Can problems be discussed and solved constructively with your colleagues? | 1 Rarely – 2 Partly – 3 Mostly – 4 Always |
| Component 2: Interprofessional organization | |
| Are tasks in your working area clearly defined? | 1 No – 2 Partly – 3 Mostly – 4 Yes, completely |
| Are meetings efficient and well structured? | 1 No – 2 Partly – 3 Mostly – 4 Yes, completely |
| Are handover talks at the change of shift efficient and well structured? | 1 No – 2 Partly – 3 Mostly – 4 Yes, completely |
| Component 3: Overall IPC | |
| Are you treated by colleagues from other wards/ other departments as you wish? | 1 Rarely – 2 Partly – 3 Mostly – 4 Always |
| Are you treated by colleagues of other professions as you wish? | 1 Rarely – 2 Partly – 3 Mostly – 4 Always |

Table 3: Main dependent variables (PRO items)

| Outcome | Item | Scale |
| --- | --- | --- |
| Overall satisfaction | What is your overall satisfaction of the treatment and care you/ your child received? | 1 Poor – 2 Moderate – 3 Good – 4 Very good – 5 Excellent |
| Less discomforts | Have the discomforts that led to your/ your child’s hospital stay improved? | 1 No, even worsened – 2 No, remained the same – 3 Yes, improved |
| Complications | Did complications occur after your/ your child’s discharge from hospital? | 1 Yes, readmission to hospital necessary due to complications – 2 Yes, treatment of complications by general practitioner/ specialist necessary – 3 Yes, but no medical treatment necessary – 4 No |
| Treatment success | How do you assess the success of medical treatment? | 1 Poor – 2 Moderate – 3 Good – 4 Very good – 5 Excellent |
| Willingness to recommend | Would you recommend this hospital to your family or friends? | 1 No – 2 Yes, probably – 3 Yes, sure |
