# Supplementary material for: Interprofessional collaboration and patient-reported outcomes: a secondary data analysis based on large scale survey data
Source: BMC Health Serv Res. 2023 Jan 3;23:5. doi: 10.1186/s12913-022-08973-5 (PMC9809039; doi:10.1186/s12913-022-08973-5)

**Additional file 3: Prespecified model for PCA**

Table 1: Items selected for PCA (independent variables)

| Description | Label | Item | Scale |
| --- | --- | --- | --- |
| Definition of tasks | QE28 | Are tasks in your working area clearly defined?* | Yes, completely – mostly– partly – no |
| Trust in colleagues | QE34 | Do you trust the colleagues, you work with directly?* | Yes, completely – mostly– partly – no |
| Support from colleagues | QE35 | Do you get necessary support from your colleagues?* | Always – mostly – partly – rarely – not necessary |
| Interaction within ward | QE36 | Are you treated by colleagues in your own ward/ your department as you wish? | Rarely – partly – mostly – always |
| Interaction between wards | QE37 | Are you treated by colleagues from other wards/ other departments as you wish? | Rarely – partly – mostly – always |
| Discussing problems | QE38 | Can problems be discussed and solved constructively with your colleagues? | Rarely – partly – mostly – always |
| Qualification of colleagues | QE39 | Do you think that your colleagues are well qualified in your working area?* | Yes, completely – mostly– partly – no |
| Distribution of tasks | QE40 | Are tasks in your working area distributed fairly?* | Yes, completely – mostly– partly – no |
| Interaction between professions (overall) | QE41 | Are you treated by colleagues of other professions as you wish? | Rarely – partly – mostly – always |
| Frequency of communication problems | QE42 | How often are there communication problems with other employees with regard to a patients’ treatment? | Very often – often – partly – rarely – not one of my tasks |
| Tasks of other responsibility | QE43 | Do you need to perform tasks that are not part of your actual field of responsibility?* | Rarely – partly – often – very often |
| Frequency of uncertainties regarding responsibility | QE44a | How often are there any uncertainties as to who is responsible for which patient? | Very often – often – partly – rarely – Not judgable |
| Interaction between doctors and nurses | QE45_1 | How well does the communication and coordination between doctors and nurses work? * | Very good – good – moderate – bad – not important – Not judgable (per question) |
| Efficient meetings | QE47_1 | Are meetings efficient and well structured?* | Yes, completely – mostly– partly – no – Not judgable |
| Efficient handovers | QE47_2 | Are handover talks at the change of shift efficient and well structured?* | Yes, completely – mostly– partly – no – Not judgable |
| Ethical conflicts | QE_UKF51 | In case of ethical conflicts, will they be discussed in a team?* | Always – mostly – partly – rarely – there are no ethical conflicts in my working area |
| Acceptance of diversity | QE_UKF52 | Is diversity accepted by employees in your working area (e. g. skin colour, religion, nationality, sexual orientation)? * | Yes, completely – mostly– partly – no |

* inverted scale

Figure 1: Prespecified model for PCA


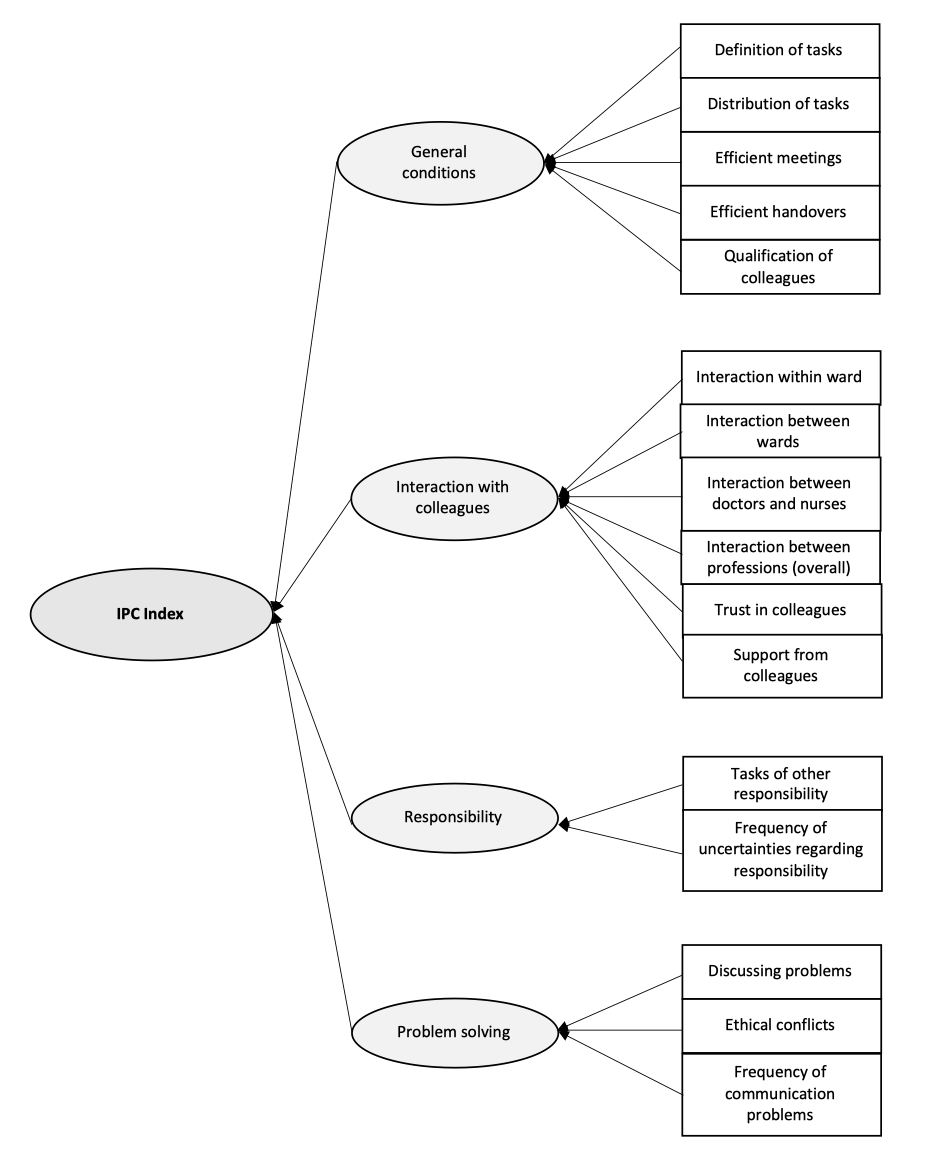

Supplement: Supplementary file 3 — Additional file 3. Prespecified Model for PCA. [file 12913_2022_8973_MOESM3_ESM.docx]
