# Supplementary material for: Interprofessional collaboration and patient-reported outcomes: a secondary data analysis based on large scale survey data
Source: BMC Health Serv Res. 2023 Jan 3;23:5. doi: 10.1186/s12913-022-08973-5 (PMC9809039; doi:10.1186/s12913-022-08973-5)
Supplement: Supplementary file 4 — Additional file 4. Survey characteristics. [file 12913_2022_8973_MOESM4_ESM.docx]

**Additional file 4: Survey characteristics**

Table 1: Survey characteristics of included hospitals

| **Hospital No.** | **Survey year(s)** | **Department type(s)** | **No. of patients (in total)** | **No. of employees (in total)** |
| --- | --- | --- | --- | --- |
| 1 | P: 2011, 2014  E: 2011, 2015 | 1, 3, 4, 7, 12, 28, 43 | 586 | 835 |
| 2 | P: 2010, 2013  E: 2010, 2013 | 1, 6, 28 | 294 | 306 |
| 3 | P: 2009  E: 2009 | 13, 15, 37, 38, 84 | 339 | 235 |
| 4 | P: 2010  E: 2010 | 1, 3, 4, 7, 63 | 243 | 376 |
| 5 | P: 2014  E: 2015 | 1, 4, 6 | 148 | 227 |
| 6 | P: 2013  E: 2014 | 2, 7, 8, 11, 12, 14, 28, 43, 58 | 344 | 501 |
| 7 | P: 2010  E: 2009 | 1, 11, 58 | 128 | 230 |
| 8 | P: 2010  E: 2010 | 1, 3, 4, 6, 14, 43 | 263 | 242 |
| 9 | P: 2010  E: 2010 | 6, 33 | 68 | 38 |
| 10 | P: 2013, 2014, 2015  E: 2014 | 5, 7, 8, 9, 10, 11, 12, 14, 16, 17, 21, 28, 31, 35, 43, 44 | 1878 | 1014 |
| 11 | P: 2011  E: 2010 | 1, 6, 43 | 150 | 73 |
| 12 | P: 2011  E: 2010 | 1, 6, 14 | 168 | 67 |
| 13 | P: 2014  E: 2015 | 1, 6 | 100 | 91 |
| 14 | P: 2014  E: 2015 | 1, 6 | 106 | 114 |
| 15 | P: 2014  E: 2014 | 1, 3, 7, 8, 11 | 236 | 111 |
| 16 | P: 2009, 2011, 2013, 2015  E: 2010, 2012, 2015 | 7, 40 | 518 | 729 |
| 17 | P: 2011  E: 2011 | 1, 4, 7, 8, 9, 11, 12, 14, 35, 58 | 420 | 381 |
| 18 | P: 2011  E: 2011 | 6, 28 | 66 | 39 |
| 19 | P: 2011  E: 2011 | 1, 6 | 99 | 101 |
|  | **P: 2009-2015 (n=26)**  **E: 2010-2015 (n=23)** | **30** | **6154** | **5710** |

E = Employee survey, P = Patient survey, 1 = General surgery, 2 = Visceral surgery, 3 = Trauma surgery, 4 = Vascular surgery, 5 = Neurosurgery, 6 = Internal medicine, 7 = Cardiology, 8 = Gastroenterology, 9 = Hematology/ Oncology, 10 = Nephrology, 11 = Gynecology, 12 = Urology, 13 = Orthopedics, 14 = Neurology, 15 = Rheumatology, 16 = Otolaryngology, 17 = Ophthalmology, 21 = Thoracic surgery, 28 = Geriatrics, 31 = Dermatology, 33 = Psychosomatics/ Psychotherapy, 35= Radiotherapy/ Radiooncology, 37 = Spinal orthopedics, 38 = Rheumatic orthopedics, 40 = Diabetology, 43 = Pediatrics, 44 = Pediatric surgery, 58 = Orthopedics and trauma surgery, 63 = Radiology, 84 Pediatric rheumatology
