# Supplementary material for: Interprofessional collaboration and patient-reported outcomes: a secondary data analysis based on large scale survey data
Source: BMC Health Serv Res. 2023 Jan 3;23:5. doi: 10.1186/s12913-022-08973-5 (PMC9809039; doi:10.1186/s12913-022-08973-5)
Supplement: Supplementary file 5 — Additional file 5. Exploratory Analyses. [file 12913_2022_8973_MOESM5_ESM.docx]

**Additional file 5: Exploratory Analyses**

Table 1: Results from adjusted exploratory analyses (n (S)= 1698; n (IM)= 2100; n (P)= 442; *n (R)= 1914))*

|  | **Overall satisfaction** | **Less discomforts** | **Complications** | **Treatment success** | **Willingness to recommend** |
| --- | --- | --- | --- | --- | --- |
| **Department-specific IPC** | S: OR 1.11 (0.97-1.27), p= 0.128, q= 0.12  **IM: OR 1.30 (1.03-1.64), p= 0.029, q=0.04**  **P: OR 0.64 (0.61-0.66), p= 0.000, q=0.001**  **R: OR 0.81 (0.69-0.96), p= 0.016, q=0.26** | **S: OR 1.17 (1.02-1.34), p= 0.026, q= 0.038**  **IM: OR 1.34 (1.12-1.60), p= 0.001, q=0.003**  **P: OR 0.30 (0.27-0.34), p= 0.000, q=0.001**  R: OR 1.41 (0.93-2.13), p= 0.110, q=0.107 | **S: OR 1.11 (1.04-1.19), p= 0.003, q= 0.007**  IM: OR 1.03 (0.93-1.15), p= 0.536, q=0.314  **P: OR 1.15 (1.07-1.23), p= 0.000, q=0.001**  R: OR 0.97 (0.80-1.17), p= 0.758, q=0.404 | **S: OR 1.12 (1.05-1.20), p= 0.001, q= 0.003**  **IM: OR 1.28 (1.04-1.58), p= 0.022, q=0.034**  **P: OR 0.48 (0.44-0.52), p= 0.000, q=0.001**  R: OR 0.96 (0.77-1.18), p= 0.681, q=0.372 | **S: OR 1.14 (1.05-1.24), p= 0.001, q= 0.003**  **IM: OR 1.30 (1.04-1.63), p= 0.020, q=0.032**  **P: OR 0.67 (0.62-0.71), p= 0.000, q=0.001**  R: OR 0.96 (0.80-1.16), p= 0.690, q=0.372 |
| **Interprofessional organization** | S: OR 0.89 (0.76-1.04), p= 0.142, q= 0.131  IM: OR 0.90 (0.66-1.22), p= 0.484, q=0.3  **P: OR 1.27 (1.12-1.44), p= 0.000, q=0.001**  **R: OR 1.29 (1.09-1.53), p= 0.003, q=0.007** | S: OR 1.01 (0.84-1.21), p= 0.900, q= 0.46  **IM: OR 0.79 (0.63-1.00), p= 0.048,** q=0.058  **P: OR 1.94 (1.58-2.38), p= 0.000, q=0.001**  R: OR 0.74 (0.52-1.06), p= 0.105, q=0.107 | S: OR 1.00 (0.83-1.02), p= 0.106, q= 0.107  IM: OR 0.88 (0.75-1.05), p= 0.151, q=0.136  **P: OR 0.59 (0.44-0.79), p= 0.000, q=0.001**  **R: OR 1.20 (1.04-1.40), p= 0.015, q=0.026** | S: OR 0.94 (0.85-1.05), p= 0.264, q= 0.227  IM: OR 0.96 (0.74-1.24), p= 0.752, q=0.404  **P: OR 1.73 (1.36-2.20), p= 0.000, q=0.001**  R: OR 1.05 (0.87-1.28), p= 0.606, q=0.328 | S: OR 0.96 (0.84-1.11), p= 0.588, q= 0.328  IM: OR 0.81 (0.65-1.02), p= 0.067, q=0.072  P: OR 1.09 (0.86-1.37), p= 0.483, q=0.3  R: OR 1.08 (0.93-1.26), p= 0.303, q=0.233 |
| **Overall IPC** | S: OR 1.01 (0.92-1.11), p= 0.89, q= 0.46  IM: OR 0.94(0.73-1.20), p= 0.603, q=0.328  **P: OR 0.74 (0.66-0.82), p= 0.000, q=0.001**  **R: OR 1.19 (1.06-1.34), p= 0.003, q=0.007** | S: OR 0.91 (0.72-1.16), p= 0.455, q= 0.3  IM: OR 0.89 (0.75-1.06), p= 0.187, q=0.162  **P: OR 0.44 (0.41-0.48), p= 0.000, q=0.001**  R: OR 1.05 (0.87-1.27), p= 0.593, q=0.328 | S: OR 1.03 (0.96-1.12), p= 0.380, q= 0.272  **IM: OR 1.17 (1.04-1.31), p= 0.007, q=0.013**  **P: OR 0.46 (0.36-0.57), p= 0.000, q=0.001**  **R: OR 1.14 (1.01-1.29), p= 0.029, q=0.04** | S: OR 0.95 (0.84-1.07), p= 0.377, q= 0.272  IM: OR 0.92 (0.73-1.16), p= 0.467, q=0.3  **P: OR 0.68 (0.57-0.81), p= 0.000, q=0.001**  R: OR 1.07 (0.91-1.26), p= 0.390, q=0.272 | **S: OR 0.90 (0.85-0.95), p= 0.001, q= 0.003**  IM: OR 0.89 (0.65-1.22), p= 0.464, q=0.3  **P: OR 0.80 (0.71-0.90), p= 0.000, q=0.001**  R: OR 1.09 (0.99-1-21), p= 0.078, q=0.082 |

S= Surgery, IM= Internal medicine, P= Pediatrics, R= Remaining departments; results are presented in OR with 95% CI, significant results are bold
